# Studies on the Enzymatic Degradation Process of Epoxy-Polyurethane Compositions Obtained with Raw Materials of Natural Origin

**DOI:** 10.3390/molecules29235667

**Published:** 2024-11-29

**Authors:** Anna Sienkiewicz, Piotr Czub

**Affiliations:** Department of Chemistry and Technology of Polymers, Faculty of Chemical Engineering and Technology, Cracow University of Technology, Warszawska Str. 24, 31-155 Kraków, Poland; piotr.czub@pk.edu.pl

**Keywords:** biodegradation, biopolymers, epoxy resins, soybean oil, wood

## Abstract

Along with the development of technology and the increasing consumption of polymeric materials, which have become an integral part of man’s everyday life, problems related to their disposal are arising. The presented research concentrates on the studies on the enzymatic degradation of selected epoxy-polyurethane materials filled with 2 or 5 wt.% of waste unmodified or chemically modified through mercerization wood flour. Composites, subjected to the degradation process, contained up to 60% of raw materials of natural origin. The enzymatic degradation was carried out for 28 days, in three environmental conditions, differing in the type of applied buffer, pH, process temperature, the amount, and the type of applied enzyme. In this study, the influence of two lipases was tested (specifically: lipase of microbiological origin—*Rhizopus Oryzae* Lipase, and one of animal origin—*Porcine Pancreas* Lipase). There were seven compositions tested, based on the polyaddition product of epoxidized soybean oil with bisphenol A, differing in the amount of filler and the type of modification to which wood flour was subjected before the application in the polymer composite. After enzymatic degradation, the greatest progress of biodegradation was observed at T = 30 °C, in a complex phosphate buffer with pH = 6.8, in the presence of the *Porcine Pancreas* enzyme. Under these conditions, a slightly smaller effect was also observed in the presence of the *Rhizopus Oryzae* enzyme. At the same time, the compositions containing mercerized wood flour turned out to be the most susceptible to biodegradation with the above-mentioned enzymes. After conducting the process in the full 4-week cycle numerous changes were noticed within the tested sample, such as (1) 7.0 %wt. of the overall weight loss of samples, (2) reducing the value of the static contact angle (e.g., from 116.7° before degradation to 27.2° at the end of the study), and (3) morphological appearance of the sample (sample’s surface had suffered erosion noticed as smoothest roughnesses and numerous empty holes throughout its entire volume), concerning sample’s condition before enzymatic degradation.

## 1. Introduction

Over the last few decades, polymer materials have become an integral part of our everyday life, finding application in many areas, such as industry, medicine, packaging, construction, and much more. Their wide range of applications results from their physicochemical properties and therefore relatively easy replacement of other commonly used raw materials, such as wood, glass, or metal. However, with the development of technology and the increasing consumption of polymeric materials, problems related to their disposal also arise [1]. In general, most synthetic polymers are characterized by low sensitivity to the influence of atmospheric factors, and biological decomposition. Particularly problematic is the utilization of waste materials made of duroplastic materials, e.g., epoxy resins. Hence, unfortunately, the increasing amount of accumulated plastic waste in the ground, rivers, and oceans, or the growing threat of global warming, as a consequence of carbon dioxide emission upon burning of non-biodegradable polymers, has increased significantly in recent years [2]. The research results are terrifying. They show that the amount of microplastics entering the ocean every year is over 3 million tonnes and, according to forecasts, unless specific action is not going to be taken, the accumulation of plastics in the ocean will exceed the mass of fish by the year 2050 [3]. Sadly, despite many pro-ecological campaigns, it seems that there is still little awareness of the long-term consequences of such human misconduct. With the development of the polymer industry, the amount of waste also constantly increases and becomes a serious ecological problem around the world. On one side such waste constitutes an ever-increasing percentage of municipal waste, while the demand for polymeric materials in Europe and around the world is constantly increasing [4]. Hence, it is extremely important to preserve fossil resources and reduce pollution. Therefore, particular attention should be paid to striving for energy-saving technologies and the production of materials using renewable resources. Studies show there are two main approaches to the biodegradability phenomenon towards minimizing the environmental impact of introducing novel polymeric materials into the market [5]. The first of them focuses on maximally extending the usefulness of the material by improving its physicochemical performance, which ideally will remain unchanged over time and exploitation. Moreover, the synthesis of such materials will involve renewable resources [6,7]. The second approach concentrates on the production of polymeric materials for short-term use that are fast biodegradable [8,9]. However, ecological awareness seems to have a great impact on combining the above-mentioned approaches by the synthesis of polymeric materials with controlled life spans. Therefore, various actions towards the disposal of plastics are undertaken [10]. First of all, more and more advanced studies are being carried out on the biodegradability of the most frequently used polymers [11]. These activities mainly involve modifying the structures of molecules to finally obtain the physicochemical properties of polymers enabling their reuse and their degradation using biological agents. In this context, the susceptibility of polymeric materials to enzymatic degradation is becoming more and more important. The susceptibility of synthetic polymers to enzymes is closely related to the physicochemical properties of the material, including its chemical composition and main chain structure. Linear polymers with a relatively simple carbon chain and low molecular weight will show much greater sensitivity to enzymatic degradation than branched, hetero-chain polymers and cross-linked form materials with large molecular weight [12].

Enzymatic degradation of polymeric materials is generally conducted using enzymes from the hydrolase group, including lipases (from the genus *Candida antarctica*), proteases (papain, bromelain), or esterases (cholesterol esterase, *Pseudomonas sp*. esterase). The most frequently described in the literature class of enzymes capable of degrading polymers are lipases. These are water-soluble enzymes that hydrolyze substrates that are insoluble in water, resulting in the reaction taking place at the phase boundary. In an aqueous environment, lipases hydrolyze triglycerides to glycerol, diacylglycerol, monoacylglycerol, and fatty acids. Lipases differ in their physical properties, thermostability, and substrate specificity. For example, an enzyme of bacterial origin is more stable than animal origin. One of the more common biocatalysts used in industry is *Porcine Pancreas* lipase (ePP), whose action is focused on the hydrolysis of fatty acid esters. During this reaction, the enzyme mainly attacks the ester bonds at external carbon atoms of glycerol, which is why *Porcine Pancreas* lipase is called also 1,3-regiospecific lipase. According to Li et al. [13], the optimal temperature for the free enzyme is 35 °C, while in the case of an enzyme immobilized with three-dimensional poly(vinyl alcohol) nanocomposite containing graphene oxide and magnetite, the optimal temperature is 40 °C. Additionally, isolated *Porcine Pancreas* lipase shows the greatest activity in the pH range of 7.5–8.5, while in the body, as a result of the presence of bile salts, this range is slightly lower (6.5–7.5 pH) [14]. Another example of an enzyme from the hydrolase group is lipase from the fungus *Rhizopus Oryzae* (eRO). This is a species of filamentous microfungus found, among others, in decomposing biomass. The enzyme *Rhizopus Oryzae* can function at temperatures up to 40 °C and in the pH range of 4.0–9.0 [15]. According to Hiol et al. [16], the optimal environmental temperature for hydrolysis induced by the isolated enzyme is 35 °C, while the highest activity of the enzyme was recorded at a pH of 7.5. Some other studies show that, during the process of deracemization of benzoin in the presence of eRO, the highest yield (73–76%) was obtained at pH = 7.5–8.5 [17].

The speed of the enzymatic degradation process depends primarily on the physicochemical properties of the polymeric material, including its chemical composition or structure of the main chain, activity, and stability of the enzyme used, and, in the case of composites, depending on the applied filler.

Generally, based on the conducted research, it was found that the lower the molecular weight of the polymer, the higher the rate of enzymatic degradation, while at the same time linear and heterochain polymers are more susceptible to degradation. In turn, taking into account the susceptibility of the polymeric material to enzymatic degradation from the point of view of the functional groups included in its composition, the susceptibility to biodegradation increases the content of groups in the series: ester > ether > amide > urethane > hydroxyl groups. It is also worth emphasizing that the enzymatic degradation of polymeric materials is favored by the amorphous rather than the crystalline structure, and also by hydrophilicity and a greater degree of specific surface area development [18,19].

One way to increase the susceptibility of a polymer material to enzymatic degradation is the addition of a filler of natural origin, e.g., starch or lignocellulosic waste. These materials, thanks to their hydrophilic nature and presence in their structure of ester and ether bonds, or numerous hydroxyl groups, can undergo enzymatic hydrolysis [20,21]. During the degradation process, more than just filler decomposition occurs, but also the weakness of the adhesion between the additive and the polymer matrix, and in consequence deterioration of the mechanical properties of the material and facilitates enzyme access to the deeper layers of the polymer.

The aim of the undertaken studies was the determination of the susceptibility for enzymatic degradation of selected epoxy-polyurethane compositions filled with waste wood flour. The research focused on the following: conducting the controlled biodegradation of epoxy-polyurethane materials in selected conditions, the analysis of the progress of the enzymatic degradation process, and finally the determination of the biodegradation conditions ensuring the greatest progress of the process.

## 2. Results and Discussion

The crosslinked epoxy-polyurethane compositions based on polyaddition products of epoxidized soybean oil and bisphenol A were subjected to enzymatic biodegradation. The polyaddition process was conducted in bulk on the course of the green modification of a well-known method of the synthesis of high-molecular-weight epoxy resins, namely ‘the epoxy fusion process’ [22,23,24]. Wood flour was introduced in an amount of 2 or 5 wt.%, based on the total weight of the composition, to the ESBO_BPA composition in the form of unmodified oak wood waste, or mercerized, using 5 or 10% solution of NaOH, oak wood waste. The choice of the concentration of the alkali solution, used for the chemical modification of wood particles was based on the results of previous studies. The increase in the concentration of the alkali solution results in the decrystallization of cellulose, increased reduction in the content of the hydroxyl groups as well as the removal of various impurities, including waxy substances of the bio filler. Hence, usually results in better adhesion of the natural filler to the polymer matrix due to better surface wetting. Chemical modification of wood waste was performed, according to the procedure described in our previous manuscript along with the analysis of the properties of modified filler and obtained epoxy-polyurethane/wood composites [23]. It is worth highlighting that the obtained epoxy-polyurethane composites, depending on the used amount of wood flour, contained 57% or 60% of raw materials of natural origin.

**Figure 1:**
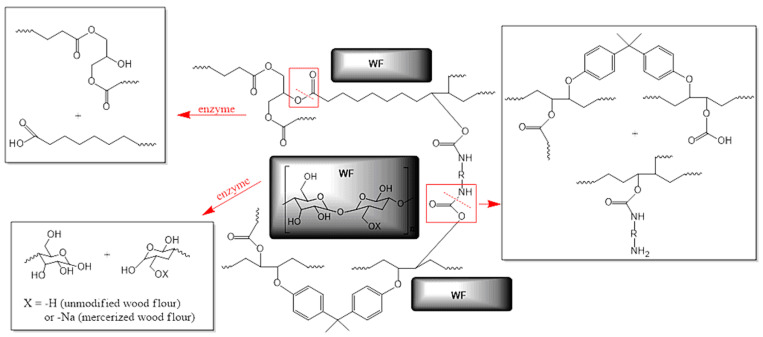
The structure of cross-linked epoxy-polyurethane compositions based on ESBO_BPA filled with unmodified and modified wood flour presented with an indication of possible degradation pathways under the influence of the enzyme (marked as red arrows; bonds marked in red boxes are susceptible to enzymatic hydrolysis).

Sustainability to enzymatic degradation of epoxy-polyurethane composites was tested on seven compositions (Table 1), which as mentioned above differ in filler content and the type of chemical modification to which the wood flour was subjected before using it as an addition to the polymer matrix. It is worth emphasizing that compositions subjected to biodegradation were in the cured form, but with elastic properties, resulting from the content of long alkyl chains of triglyceride molecules and relatively low cross-linking density compared to BPA-based petrochemical epoxies. Additionally, these materials contain large amounts of ester and urethane bonds, so that in the purposes of studies on the biodegradation process we chose lipases from the group of hydrolases. Figure 1 presents the general structure of composites subjected to enzymatic degradation with the indication of bonds, which are especially sensitive to the action of applied enzymes.

**Figure 2:**
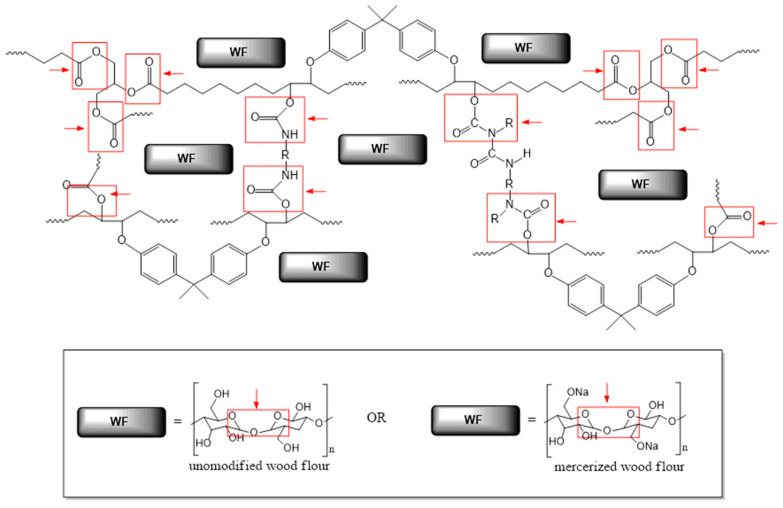
Probable breakdown of the epoxy-polyurethane composition upon the enzymatic degradation (red arrows represent the action of enzymes; while bonds marked in red boxes are susceptible to enzymatic hydrolysis).

The main role of these enzymes concentrates on breaking ester bonds. However, numerous scientific publications [20,25,26] also highlight their ability for urethane bond decomposition (Figure 2).

The compositions were degraded using the enzymes *Porcine Pancreas* lipase (ePP) and *Rhizopus Oryzae* lipase (eRO). Both enzymes are from the lipase family and are capable of hydrolyzing ester linkages to form products containing hydroxyl groups, amine derivatives, and CO_2_. To carry out the biodegradation process, three phosphate buffers based on sodium hydrogen phosphate and potassium dihydrogen phosphate were prepared. Biodegradation was carried out in three different environmental conditions for 28 days, with weekly environmental and enzyme exchange for fresh. The samples were removed from the vials, washed with methanol, and then further dried in a laboratory dryer at 60 °C for approx. 1 h. After this time, the degraded samples were analyzed for changes in mass, contact angle, and chemical structure.

### 2.1. Analysis of the Change in Mass of Composite Samples Degraded in the Presence of Enzymes

**Figure 3:**
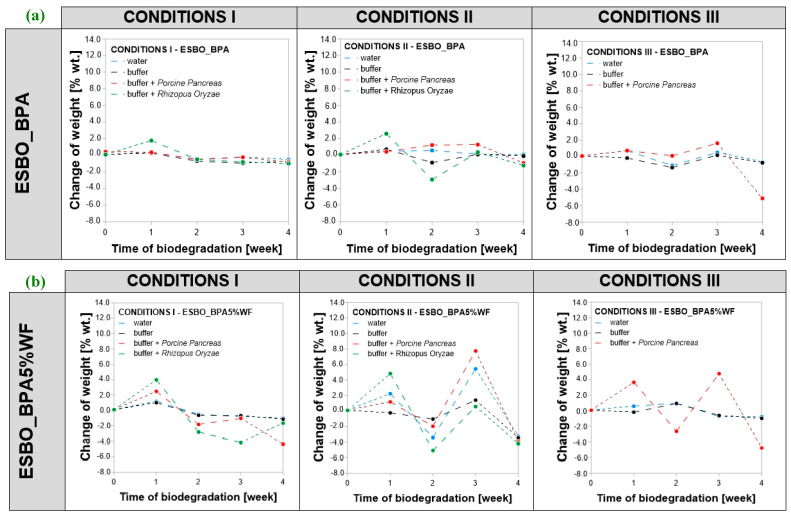

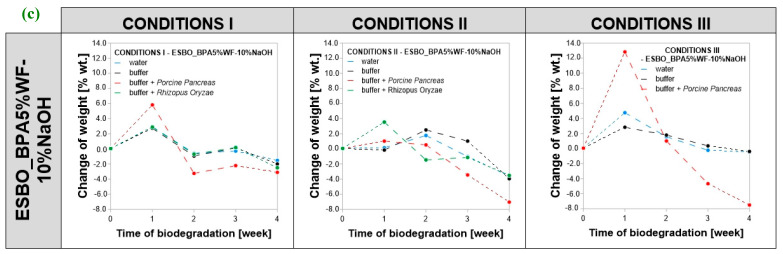
(**a**–**c**). Change in the mass of bio-composite samples based on ESBO_BPA depending on the conditions of enzymatic degradation: (1) conditions I—T = 37 °C, pH = 7.3, in a KH_2_PO_4_/Na_2_HPO_4_ phosphate buffer environment; (2) conditions II (T = 30 °C, pH = 6.8, in complex phosphate buffer environment), and (3) conditions III—T = 40 °C, pH = 7.5, in a KH_2_PO_4_/Na_2_HPO_4_.

For all samples subjected to biodegradation, a change in mass concerning initial value was recorded (Figure 3). The analysis of changes in the mass was considered as the percent of weight loss of samples (marked as %wt.) and it was calculated as ${∆m}_{week\;number}=\frac{m_{f}-m_{0}}{m_{0}}\cdot 100\%$, where ${∆m}_{week\;number}$—samples’ weight change in each week, $m_{f}$—samples’ measured weight in a given week, and $m_{0}$—weight of a sample before degradation. Comparing the obtained results, it was found that the greatest changes in the mass were recorded for samples incubated in conditions II. The overall recorded weight loss after conducting the process in the full 4-week cycle was at the level of −7.0 %wt. (ESBO_BPA5%WF-10%NaOH), concerning the weight of the samples before enzymatic degradation. In turn, in the case of conditions I and III, a weight loss also was recorded; however, the change was smaller than for samples degraded in conditions II with a maximum of −4.7 percentage weight loss (ESBO_BPA5%WF). Simultaneously, the greatest mass changes were recorded for samples incubated in the presence of the *Porcine Pancreas* enzyme regardless of the conditions, including the type of buffer and incubation temperature, in which the degradation was carried out. In turn, in the case of samples degraded in the presence of the *Rhizopus Oryzae* enzyme, the samples’ weight changes were smaller than in the case of samples incubated in the presence of the *Porcine Pancreas* enzyme. Based on the obtained results, it can be assumed that in the degradation of epoxy-polyurethane wood composites based on epoxidized soybean oil the *Porcine Pancreas* enzyme exhibits a greater ability for hydrolyzing chemical bonds within the structure of degraded samples, especially when using a complex mixture buffer solution PB2, temperature 30 °C and pH equal to 6.8.

Considering the behavior of samples of each type of analyzed compositions (ESBO_BPA, ESBO_BPA5%WF, and ESBO_BPA5%WF-10%NaOH), it was noticed that for samples of cured ESBO_BPA the largest total overall weight change was observed for degradation in condition III, in which the average weight loss in the presence of *Porcine Pancreas* was—5.1 %wt. After the incubation of samples under condition I, there was just a slight reduction in the samples’ weight. It was about −1.0 %wt., regardless of the application of enzyme and incubation solution. For samples incubated for 4 weeks in condition II, the weight loss was on the level of −1.2 %wt. for samples soaked with the *Rhizopus Oryzae* enzyme and −0.95 %wt. for those with *Porcine Pancreas*, respectively. Within the first week of conducting the process in the presence of eRO, both in conditions I and II, an increase of about +2 %wt. of samples was recorded, while after the second week, the weight loss was recorded (−0.5 % wt.—condition I, and −2.9 %wt.—condition II, respectively). In turn, for samples incubated with ePP, regardless of conditions of the degradation process through 3 weeks of degradation a slight change in weight was discovered, usually it was about +0.3–0.7 %wt. increase with a small drop of weight in the second week (−0.5 %wt.—condition I and −0.1 % wt.—condition III) or further increase in weight by about +1.2 %wt. in the second week and +1.3 %wt. in the third week of degradation, concerning the initial weight of the samples.

For samples with wood filler for the first week of degradation, an increase in weight was discovered and it was much more noticeable than in the case of unfilled composition of ESBO_BPA (from +4.0 to +12.8 %wt.). This is probably due to the presence of hydrophilic lignocellulosic filler in composites, which during the second week was probably leached from the composition due to poor bonding with the polymer matrix. In general, such leaching in the second week was the most visible in the case of unmodified wood filler. Next, probably after some portion of wood filler was removed from the matrix, the fresh incubation solution could more easily penetrate the composite through newly formed holes and that is why, in general, for those composites in the third week an increase in weight was recorded. Considering the behavior of ESBO_BPA5%WF and ESBO_BPA5%WF-10%NaOH samples during degradation, concerning the behavior of pure composition of ESBO_BPA during that time, it was found that within the first week in all samples an increase in weight is observed. The significant differences concern the second and the third week of degradation. Usually within the second week, a significant drop in weight was observed, while in the third week, a significant increase in weight was recorded again. Only in the case of samples ESBO_BPA5%WF in conditions I and eRO, and composition with mercerized wood filler in conditions II and III, incubated with ePP, was further weight loss noticed. The observed weight gain of all samples was caused by the phenomenon of swelling of both the polymer matrix and filler in the environment of incubation solutions. It is worth highlighting that such swelling of the sample subjected to the degradation process is a desirable phenomenon, since in the case of degradation of polymeric materials, the intensity of degradation depends, among other things, on obtaining the largest possible contact area of the macromolecular material with applied degradation factors. Hence, wood filler in composites subjected to enzymatic degradation played an important role, especially in the introduction stage of biodegradation. First of all, due to greater than the polymer matrix’s tendency of wood additives to swell under the influence of the incubation solutions, wood particles in the swollen form exerted additional pressure on the bonds within the surrounding polymer matrix. Moreover, when during the degradation, the wood filler was leached out leaving behind empty spaces in the cross-linked structure of the polymer material. Hence, the actual total surface area exposed to the action of the enzymes in addition to the surface action has been increased by the free spaces left by the filler used. For all wood composites, after 28 days of conducting the process and weighing completely dried samples, a weight loss was noticed. In general, the greatest change was recorded for samples incubated with *Porcine Pancreas* enzyme (a drop of weight from −1.1 %wt. to −7.0 %wt. concerning the initial mass of dry samples before degradation). Within those samples, the biggest weight loss was recorded for ESBO_BPA5%WF-NaOH in condition II.

### 2.2. The Change in pH of Incubation Solutions During the Enzymatic Degradation of Epoxy-Polyurethane Wood Composites Based on Epoxidized Soybean Oil

**Figure 4:**
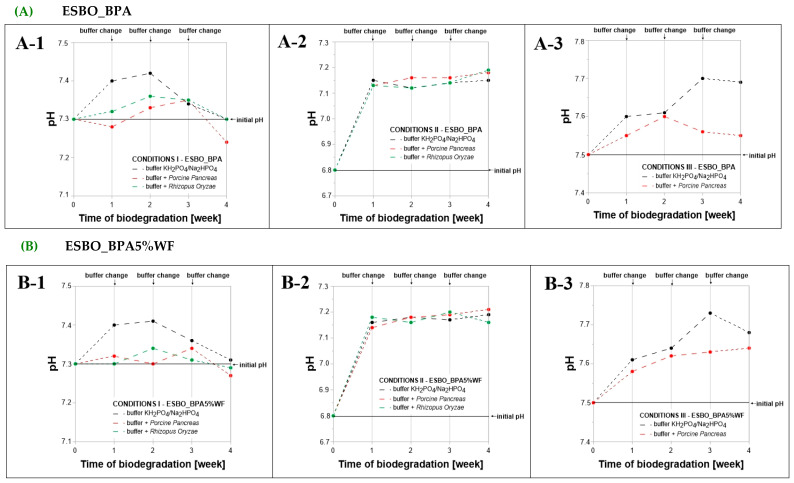

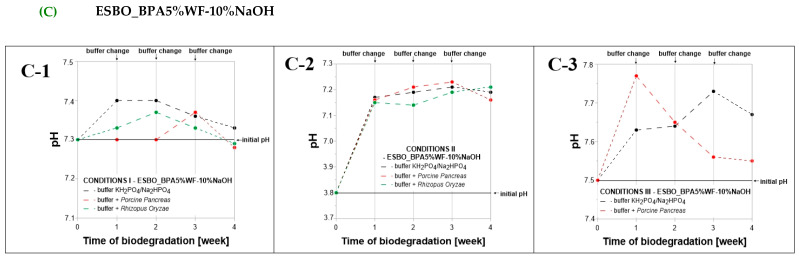
Change in pH of the incubation solutions, depending on conditions of the performed enzymatic degradation in the presence of lipases ((**A-1**–**C-1**)—conditions I: T = 37 °C, pH = 7.3, in a KH_2_PO_4_/Na_2_HPO_4_ phosphate buffer environment; (**A-2**–**C-2**)—conditions II: T = 30 °C, pH = 6.8, in complex phosphate buffer environment and (**A-3**–**C-3**)—conditions III: T = 40 °C, pH = 7.5, in a KH_2_PO_4_/Na_2_HPO_4_), and the samples subjected to degradation ((**A**)—ESBO_BPA, (**B**)—ESBO_BPA5%WF, and (**C**)—ESBO_BPA5%WF-10%NaOH).

During the enzymatic degradation using two types of lipases, the incubation solutions were replaced with fresh ones after each week of conducting the process. It was found that, depending on the conditions in which degradation of bio-composites took place, various changes in values of pH were recorded concerning the initial values measured for each phosphate-buffer solution (Figure 4). In general, each week the recoded values were higher than the pH value for the freshly applied solution. Considering single conditions through time, the course of changes, characteristic for buffer solutions without enzymes is practically the same. The differences can only be considered due to the type of enzyme applied in the incubation solution. Within condition I, after the last week of the degradation process, the biggest drop in pH value was recorded for the *Porcine Pancreas* degradation solution of ESBO_BPA (change from pH = 7.3 to pH = 7.25). Even smaller differences between solution with and without the enzyme are noticeable for condition II, for which each week the recorded increase in pH value was similar and after the four weeks of degradation the value of pH was on the level of 7.15–7.19. The biggest changes within the course of the pH changes through the process comparing the pure buffer solution and the one with ePP were observed for conditions III. Especially for samples ESBO_BPA5%WF-NaOH, in the case of the first week of incubation, a sharp increase in pH value was recorded for the enzyme solution. It is worth highlighting that, during that time for this composite, the biggest increase in the weight of samples was also noticed. Such a phenomenon may indicate that together with the swelling of samples, considered especially as swelling of its modified lignocellulosic filler, the hydrolysis of -ONa groups took place.

### 2.3. Analysis of the Change in the Static Contact Angle of the Surface of Samples Subjected to Degradation in the Presence of Porcine Pancreas and Rhizopus Oryzae Enzymes

**Figure 5:**
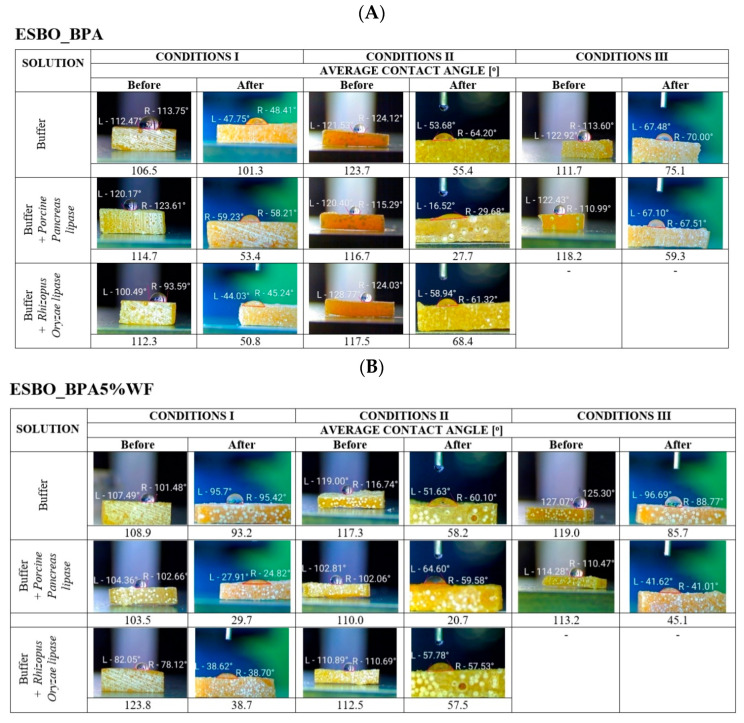

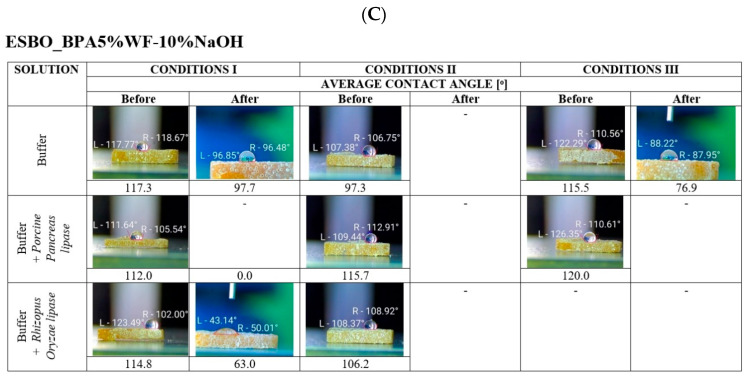
The average value of the contact angle before and after the biodegradation process. Images within the above tables show one of three samples, subjected to degradation under given conditions, while the value below represents the resultant average value. ((**A**)—ESBO_BPA, (**B**)—ESBO_BPA5%WF, and (**C**)—ESBO_BPA5%WF-10%NaOH)

Figure 5 presents photographs and average values of the static contact angle for cross-linked samples, based on the polyaddition product of epoxidized soybean oil and bisphenol A. Based on designated values of contact angle, it was found that all samples before degradation were characterized by a hydrophobic surface. The average value of the measured contact angle for samples of epoxy-polyurethane material without wood filler was 103–120° (Figure 5A). At the same time, it was observed that the introduction of wood flour into the polymer matrix resulted in a decrease in the value of contact angle. The average measured values of static contact angle, before degradation for bio-composites containing wood flour, were 90–120° (Figure 5B,C). Simultaneously, it was observed that regardless of the type of applied flour in the epoxy-polyurethane composite, the surface of samples containing 5 wt.% of filler showed greater hydrophilic properties than those with 2 wt.% of WF.

In turn, after the incubation of all samples of bio-composites in appropriate buffer solutions, and under specific degradation conditions, a change in the contact angle was found. A decrease in the hydrophobic nature of the sample surfaces was noticed for material without wood flour, which was incubated in the buffer solution itself. The measured values of static contact angle for ESBO_BPA/buffer were as follows: 100°—conditions I, 55°—conditions II, and 50°—conditions III, respectively. The addition into the buffer solution selected lipase used in the degradation process resulted in a greater increase in the hydrophilicity of the surface of ESBO_BPA samples.

The greatest effect on reducing the value of the static contact angle had the *Porcine Pancreas* enzyme in a complex phosphate buffer with pH = 6.8 at T = 30 °C (conditions II, average change in measured three values of static contact angle was from 116.7° before degradation to 27.2° at the end of the study). Simultaneously, the *Rhizopus Oryzae* lipase had the greatest effect on the surface hydrophilicity of the ESBO_BPA samples when the degradation was performed in KH_2_PO_4_/Na_2_HPO_4_ phosphate buffer with pH = 7.3 at T = 37 °C (conditions I, the average change from 112.3° to 50.8°).

Figure 5B presents the results of the studies of the influence of the degradation conditions on the static contact angle of bio-composites containing unmodified wood flour. As stated, in the Section 3 ‘Materials and Methods’, composites, which contain filler in amounts of 2 or 5 wt.%, were subjected to enzymatic degradation. Based on performed studies a larger amount of filler causes greater changes in the value of the static contact angle, especially in conditions II. It was found that the bio-composite with the addition of 5 wt.% of unmodified wood flour was characterized by even greater hydrophilicity than the bio-composite containing 2 wt.% of the natural filler. As a result of enzymatic degradation, carried out under conditions I, for samples ESBO_BPA2%WF incubated in the presence of the *Porcine Pancreas* lipase, the initially measured value of the contact angle decreased to the value of 32–38°, while in the case of application of *Rhizopus Oryzae* enzyme—to a value of 55–58°. In condition II, for samples incubated in the buffer with *Porcine Pancreas*, an average value of contact angle was 40–42°, and for the samples degraded in the presence of *Rhizopus Oryzae* enzyme, an even larger increase in the hydrophilicity was noticed (average contact angle of 22–24°). The significant influence of the enzyme *Porcine Pancreas* on the nature of the surface of the incubated samples was observed during the incubation of samples in condition III. However, in a comparison of enzyme influence under all three studied conditions, the final effect was the smallest (contact angle 55–62°). At the same time, it was found that for samples containing a larger amount of wood filler the greatest impact on changing the nature of the composite surfaces during enzymatic degradation had the *Porcine Pancreas* lipase in the environment of a complex phosphate buffer with pH = 6.8 at 30 °C (conditions II).

In the case of the degradation of composites with mercerized wood flour, it was observed that the surface of all bio-composite samples incubated in conditions II, regardless of the type of applied enzyme, changed their nature drastically. In some cases, it was even impossible to measure the value of the contact angle. The drop of distilled water, applied during the test, completely penetrated the sample almost immediately after it made contact with the surface of the sample.

The analysis of the nature of the samples’ surfaces was performed also for samples containing wood filler chemically modified using various concentrations of the alkali solution (WF-5%NaOH or WF-10%NaOH, obtained via the mercerization process with 5 or 10% solution of NaOH, respectively). Generally, it was found that samples containing mercerized wood flour, obtained by the treatment of lignocellulosic filler with 10% alkali solution, at the given share of wood flour within the composite (2 or 5 wt.%), became more susceptible to enzymatic degradation.

### 2.4. FT-IR-ATR Analysis of Composites

**Figure 6:**
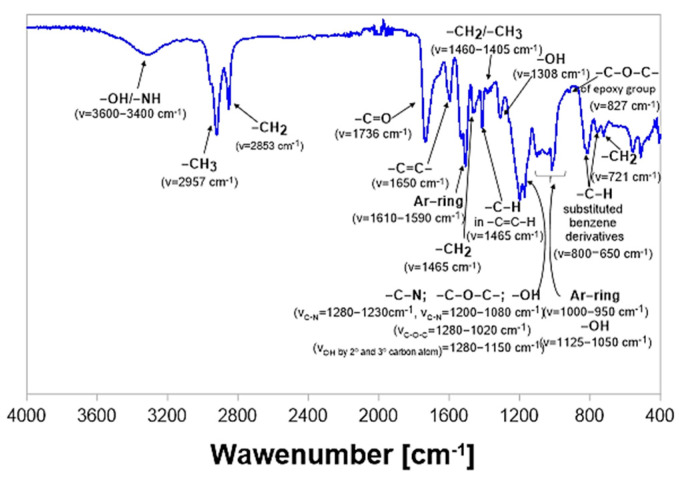
FT−IR spectrum of the sample of cured ESBO_BPA composition before enzymatic degradation.

The degradation process of samples of epoxy-polyurethane composites was also monitored by analysis of the chemical structure using the FT-IR-ATR method. Spectra were recorded before incubation, after each week of the degradation process, and finally, after a full cycle of 28 days, when samples were completely dry due to a 24 h drying process in a laboratory dryer at 60 °C. Figure 6 presents a chemical structure with a detailed description of a composition based on a polyaddition product of epoxidized soybean oil and bisphenol A cured with 4,4′-diphenylmethane diisocyanate. On the spectrum below, in the region of 3320–3520 cm^−1^, the presence of vibrations with relatively low intensity corresponding to the vibrations of -OH/-NH groups was found [27]. Next, in the area of 2820–2980 cm^−1^, a double signal originating from—CH_3_/-CH_2_ moieties was detected. In turn, in the range of 1550–1750 cm^−1^, a signal associated with the ester group was observed.

Based on the previously conducted studies [25], by the curing process using 4′-diphenylmethane diisocyanate, a reduction in the intensity in the range of 3300–3500 cm^−1^ might be detected. In this area, within the spectrum of pure non-crosslinked polyaddition products, bands corresponding to the vibrations of hydroxyl groups are present. Observed differences within the intensity of band between spectra of uncured and crosslinked polyaddition product results from the cross-linking reaction in which free hydroxyl groups of polyaddition products reacted with isocyanate groups of 4,4′-diphenylmethane diisocyanate. Moreover, the bands present in the spectrum of cured products in the range of 1695–1740 cm^−1^ correspond both to vibrations of carbonyl groups and newly formed urethane groups.

It is also worth highlighting that for all samples, tested before the degradation process and containing lignocellulosic filler, a greater intensity of vibration in the area of 3320–3520 cm^−1^ was recorded. It resulted from the larger number of -OH groups that are a part of the chemical structure of the wood flour, which was introduced into the epoxy material before its curing with diisocyanate [23].

As a result of the application of selected lipase enzymes during performed enzymatic hydrolysis, significant changes should be observed in the areas associated with the ester and urethane bonds, particularly sensitive to hydrolytic decomposition in the presence of *Porcine Pancreas* and *Rhizopus Oryzae*. Simultaneously, the expected products of such degradation among others could include amine derivatives. The progress of the enzymatic degradation in polyurethane-like compounds is usually visible as the decrease in the ratio ester (C=O)/ether (1720 cm^−1^/1125 cm^−1^), the ratio urethane (NH)/ether (1630 cm^−1^/1125cm^−1^) and the ratio aryl (C = C)/ether bond vibrations [28].

**Figure 7:**
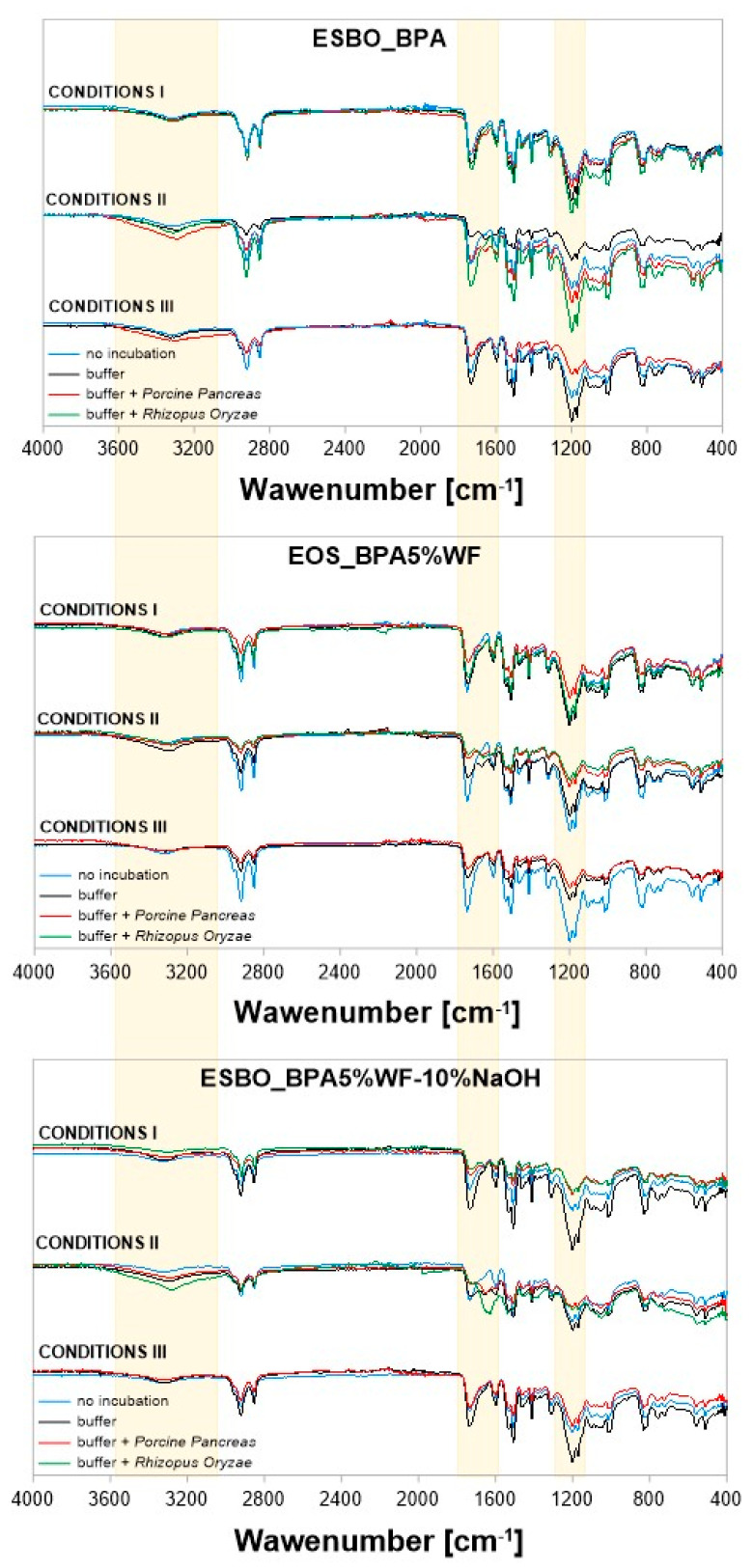
FT−IR spectra of the composite samples subjected to enzymatic degradation: ESBO_BPA, ESBO_BPA5%WF, and ESBO_BPA5%WF-NaOH.

On the FT-IR spectrum, the band characteristic for primary amines and their derivatives usually occurs in the range of 3500–3300 cm^−1^ (asymmetric and symmetric N-H stretching vibrations) and often this band overlaps with the corresponding signal of hydroxyl groups [28]. On the other hand, N-H deformation vibrations, which usually appear within the FT-IR spectrum at 1650–1500 cm^−1^, may be difficult to identify when analyzing the structure of molecules in which -C = O bonds are also present [29,30]. Generally, as a result of the performed degradation, an increase in signal intensity of characteristic vibrations -OH/-NH in the area of 3520–3320 cm^−1^ was noticed (Figure 7). However, the most visible changes were observed in the case of samples incubated in conditions II and III for samples soaked in buffer solutions of the *Porcine Pancreas* enzyme. In these conditions, an increase is particularly visible for samples ESBO_BPA.

The range of 1550–1750 cm^−1^, marked in the above spectra, is related to the presence of ester groups in the chemical structure of the analyzed samples. It was noticed that after the enzymatic degradation, the intensity of vibrations was significantly reduced for samples containing wood filler. The biggest changes are noticeable in the case of degradation in conditions II. Moreover, for those samples, the greatest changes were also recorded regarding the vibrations of the -C-O-C- group in the region of 1240–1140 cm^−1^.

Analyzing changes in the chemical structure of individual compositions containing a filler in the form of wood flour, it was noticed that in the case of the composite sample with unmodified wood flour the greatest changes are visible for samples degrading in the phosphate buffer of *Porcine Pancreas* enzyme under conditions II and III. These changes are concerned with the intensity of vibration in all areas mentioned above, especially the one characteristic for vibrations -OH/-NH in the area of 3520–3320 cm^−1^. For samples degraded in the presence of the *Rhizopus Oryzae* enzyme under conditions II, in that region, the vibration intensity was comparable with the intensity, which was recorded for samples before the process. However, for other areas of the spectrum, the registered vibration was more intensive than for samples before degradation and those observed for samples after the degradation in the presence of the *Porcine Pancreas* enzyme.

In the case of spectra recorded for samples containing mercerized wood flour, it was found that the greatest disappearance of ester groups was observed for samples degraded by the *Rhizopus Oryzae* enzyme under conditions I.

In turn, reducing the intensity of the signal characteristic of the -C-O-C- moiety in the region of 1240–1140 cm^−1^ is noticeable both as a result of samples incubation in a phosphate-buffer solution with pH = 7.3, at 37 °C, and containing the *Rhizopus Oryzae* enzyme (condition I), as well as degradation in the presence of the *Porcine Pancreas* enzyme under conditions III (pH = 7.5, T = 40 °C).

As mentioned in the beginning, within the conducted research epoxy-polyurethane compositions containing different types of wood filler were tested. Among others, we subjected to the enzymatic degradation compositions with wood filler chemically modified by mercerization using different concentrations of modifying NaOH mixture (5 or 10%, thus obtaining mercerized wood flour WF-5%NaOH or WF-10%NaOH, respectively). FT-IR spectra recorded for these samples after 4 weeks of degradation differ significantly in the region characteristic for the signal of -C-O-C- groups vibrations. Such a phenomenon is especially visible for samples that were incubated in conditions II in the presence of the *Porcine Pancreas* enzyme.

### 2.5. Analysis of Changes Within the Morphology of the Surface of Biodegraded Samples

**Figure 8:**
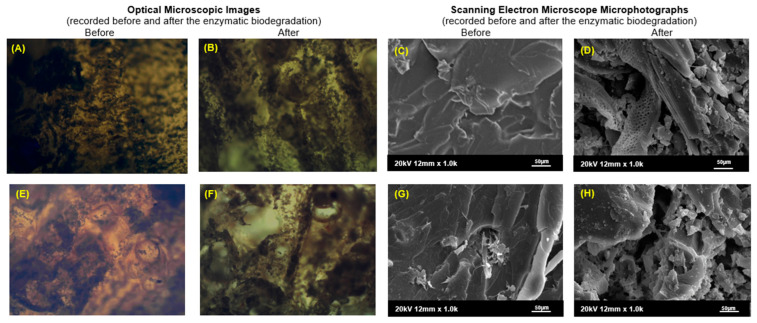
Microscopic analysis of selected samples: (**A**–**D**)—ESBO_BPA and (**E**–**H**)—ESBO_BPA5%WF-10%NaOH before and after enzymatic degradation (images A-B and E-F recorded using an optical microscope with transmitted light and 40× magnification, while (**C**,**D**) and (**G**,**H**) recorded using Scanning Electron Microscope at 5 kV acceleration with dusting the surface of samples with a thin film of gold).

Additionally, selected samples of epoxy-polyurethane compositions were subjected to microscopic analysis using an optical microscope and scanning electron microscope. Figure 8 presents images of the ESBO_BPA and ESBO_BPA5%WF-10%NaOH compositions, which were recorded before and after the degradation process in the presence of the *Porcine Pancreas* enzyme, in a complex phosphate buffer with pH = 6.8, at a temperature of 30 °C (conditions II).

All registered images of composite surfaces before the degradation process (Figure 8A,E) using the optical microscope present their roughnesses with irregular shapes and easily noticeable sharp edges. However, four weeks of incubation of samples in an enzymatic environment resulted in visible changes on their surface (Figure 8B,C). All surfaces had been eroded, whereby the samples became smoother, with less visible roughness. By analyzing the entire package of degraded samples, it can be concluded that, regardless of the condition of conducting the degradation, including the type of incubation solution, temperature, and pH of the environment, the greatest changes were observed for the samples incubated in the presence of the *Porcine Pancreas* enzyme. At the same time, the greatest changes in the morphology of the surface observed using an optical microscope are visible for the samples containing wood filler, which may indicate a greater susceptibility of these biocomposites to the erosive effect of lipases. Degradation of most of these samples led to the presence of empty holes throughout the entire volume of the degraded sample. An example of such destruction is visible in the sample presented in Figure 8F. Additionally, selected samples, for which the greatest changes were observed with the 40× magnification were also subjected to further surface analysis using a scanning electron microscope. SEM micrographs of samples after 4 weeks of incubation in the enzyme’s environment, compared to images recorded for the composition before degradation, reveal significant changes in the structure of the material. Numerous irregularities, damages, and holes are visible and they were formed as a result of the action of applied lipases. In the microphotograph of ESBO_BPA composition, similar to SEM images registered for other polymeric materials, described in the literature [30,31], an uneven surface and numerous clusters of holes in the polymer matrix were observed. Similar holes and irregularities were also found in the case of the degraded composition containing modified wood flour. In this case, it should be emphasized that larger holes could have been created as a result of leaching the wood filler that was less strongly bonded to the polymer matrix. Wood waste filler, unlike additives, such as corn starch, cocoa, green coffee, or cinnamon extracts tested in other polyurethane-like materials [32,33], subjected to enzymatic degradation increased the susceptibility of the epoxy-polyurethane material to biodegradation.

## 3. Materials and Methods

Materials. Cured epoxy-polyurethane compositions based on polyaddition product of epoxidized soybean oil and bisphenol A filled with waste wood flour (ESBO_BPA).

The synthesis of epoxy-polyurethane compositions filled with wood waste particles.

The synthesis of epoxy-polyurethane materials filled with wood waste flour was composed of several stages: (1) obtaining the polyaddition product based on epoxidized soybean oil, (2) chemical modification of wood waste flour, (3) introduction of wood filler to polyaddition product, and (4) crosslinking the obtained mixture with polyisocyanate. All stages were performed following procedures developed during previous studies [22,23,24]. In the fusion reaction epoxidized soybean oil (Ergoplast, Boryszew ERG S.A., epoxy value EV = 0.363 mol/100 g), bisphenol A (BPA, GE Cartagenie, 99.93%), and LiCl (Merck, pure; in the amount of 0.002 mol per 1 mol of bisphenol A) were used. The process was carried out in the nitrogen atmosphere. In the beginning, epoxidized soybean oil was heated to 110 °C, and a calculated amount of the BPA was added. Then, the reaction mixture was homogenized, followed by adding the LiCl and rising to the desired temperature of 160 °C. The polyaddition product, in the liquid form, was mixed mechanically with lignocellulosic filler for 30 min, followed by the addition of a defoamer (BYK A530, in the amount of 1 wt.% based on the total weight of the prepared composition) and finally the obtained epoxy composition was crosslinked using Desmodur VL (based on 4,4′-diphenylmethane diisocyanate) in a stoichiometric amount concerning hydroxyl groups in hardened material (Table 1).

Wood flour, obtained as post-production waste from parquet flooring installation, was separated into homogeneous fractions, wherein the research a fraction with a size <0.04 mm was used. Moreover, before the application of additives as a component of epoxy composites, the wood flour was predried at a temperature of 80 °C for 48 h and then subjected to mercerization. The chemical modification was carried out for 30 min, using 5% and 10% sodium hydroxide solutions. Next, the wood particles were neutralized, rinsed with distilled water, and filtered in a Büchner funnel. Finally, the modified oak waste was dried at 80 °C for 48 h, grounded, and fractionated using a laboratory shaker for sieve analysis.

Biodegradation process:

The following ingredients were used for the enzymatic degradation of epoxy-polyurethane compositions:

- Enzymes: *Rhizopus Oryzae* lipase (eRO, Sigma-Aldrich, St. Louis, MO, USA; solid, light brown powder; lipolytic food enzyme, which is produced in controlled fermentation of fungi *Rhizopus Oryzae*, an enzyme from the group of hydrolyzes, it catalyzes the ester bonds hydrolysis) and *Porcine Pancreas* lipase (ePP, Sigma-Aldrich; solid, light yellow granules; enzyme isolated from pig pancreas, catalyze the hydrolysis of ester bonds in aqueous solutions to form hydroxyl groups and carboxylic acids).
- Phosphate buffers based on sodium hydrogen phosphate and potassium dihydrogen phosphate:

(1) PB1, pH = 7.3—Na_2_HPO_4_ (C_M_ = 1 mol/L): KH_2_PO_4_ (C_M_ = 1 mol/L) = 1:3;

(2) PB1, pH = 6.8—Na_2_HPO_4_ (7.0 g/mol), KH_2_PO_4_ (3.0 g/mol), NH_4_Cl, (1.0 g/mol), MgSO_4_·7H_2_O (0.25 g/mol), NaCl (0.5 g/mol), H_3_BO_3_ (0.5 μg/mol), CuSO_4_·5H_2_O (40.0 μg/mol), FeCl_3_·6H_2_O (0.2 μg/mol), ZnCl_2_ (0.4 μg/mol), MnSO_4_·5H_2_O (0.4 μg/mol), (NH_4_)_5_Mo_7_O_24_·7H_2_O (0.2 μg/mol) and distilled water;

(3) PB1, pH = 7.5—Na_2_HPO_4_ (C_M_ = 1 mol/l): KH_2_PO_4_ (C_M_ = 1 mol/l) = 1:5.

Samples of polymeric compositions for mechanical tests in the form of beams were cut into cuboids with an average size of 0.5 cm × 1 cm × 0.2 cm and a weight ranging from 0.02 to 0.09 g. The bio-composites were then cleaned of impurities, generated during cutting, through repeated washing with methanol, dried, and initially analyzed before proper incubation in the presence of lipase enzymes. The prepared samples were placed in screw-cap glass vials with a capacity of 4 cm^3^. The degradation of the composites was carried out in three different conditions (Table 2). For each degradation, 3 samples of one composition were cut out, incubated in separate vials, and analyzed results were averaged.

Regardless of the chosen degradation conditions, in each case, the samples were incubated in given solutions for 28 days, with weekly environmental and enzyme exchange for fresh, in laboratory incubators intended for this purpose (ChemLand Laboratory Incubator WPL Series, with forced circulation 065L, range +5–120 °C). The weekly exchange of the buffer and enzyme was dictated by an attempt to maintain the activity of the enzymes at a constant level during each week of the study. After every 7th day of the entire 28 days of the conducted research, polymeric samples were analyzed to observe possible changes in the appearance of samples and their chemical structure. Parallel to enzymatic biodegradation, control tests were carried out in distilled water and without the use of an enzyme.

Methods. The tested epoxy-polyurethane compositions were subjected to a comprehensive analysis before, every 7th day of each cycle, and after the biodegradation process. The analysis of the progress of the biodegradation process was based on the monitoring of changes in the pH of the environment (pH-meter CP-40 1, pH-sensor InPro3100/120/PT100, Elmetron, Zabrze, Poland), mass (analytical weight AS R2 PLUS, Radwag, Krakow, Poland), surface morphology using an optical microscope (Delta Optical ProteOne, Delta Optical, Warsaw, Poland), and Scanning Electron Microscope (JEOL JSM-6010LA, Tokyo, Japan at 5 kV acceleration with dusting the surface of samples with a thin film of gold), contact angle (digital microscope Delta Optical Smart 2 MPix USB, Delta Optical, Poland), and chemical structure of tested materials (spectrophotometer Nicolet iS5 FTIR with a diamond attachment iD7 ATR, spectra were recorded for wavenumbers in the range of 4000–600 cm^−1^ in 4 cm^−1^ intervals, sixteen scans were averaged, ThermoFisher Scientific, Waltham, MA, USA; spectra were registered using 93 OMNIC software, ThermoFisher Scientific, USA).

## 4. Conclusions

After enzymatic degradation in the presence of lipases conducted under various conditions of the compositions based on epoxidized soybean oil filled with wood flour, it was found that the best conditions for carrying out the enzymatic degradation are conditions II (T = 30 °C, pH = 6.8), in the presence of the *Porcine Pancreas* enzyme, in a complex buffer environment. In these conditions, the greatest changes in samples’ weight, pH of the incubation solution, surface contact angle, the intensity of esters bond vibration, and changes in surface morphology were recorded. This indicates that the highest activity to degrade ester bonds included in the chemical structure of composites based on polyaddition products of natural bases presents the enzyme of animal origin.

For composite samples, which were incubated in the presence of the *Rhizopus Oryzae* enzyme under conditions II, changes in the tested parameters were also found. However, observed changes were not that significant, as in the case of the degradation carried out using the *Porcine Pancreas* enzyme.

Additionally, it was found that the presence of the filler in the form of unmodified wood flour influenced the increasing susceptibility of the bio-composite to the action of enzymes. It can be assumed that the hydrophilic nature of the lignocellulosic filler allowed for the greater penetration of the polymer material by enzymes contained in incubation solutions, which, among others, after the degradation process resulted in a reduction in the value of static contact angle for analyzed samples.

Comparing the impact of the degradation on composites containing 2 or 5 wt.% of wood flour, it can be concluded that larger changes, including, e.g., surface contact angle, were observed in the case of material containing 5 wt.% of filler. In turn, the application of chemically modified (by mercerization of raw wood flour with 5 or 10% solution of NaOH) in epoxy-polyurethane composites, resulted in recording even greater changes observed during and after the enzymatic degradation process. In the case of these composites, greater changes in the pH of incubation solutions along with simultaneously recorded greater changes in the mass of degraded samples and a significant reduction in the intensity of vibrations characteristic of ester bonds were recorded (degradation under conditions III, in the presence of *Porcine Pancreas* enzyme). For these samples, large changes were also recorded in the morphology of the surface and the associated drastic change in the value of the static contact angle. Before degradation, the surface of the samples showed typical characteristics of hydrophobic materials, and after degradation, in some cases, the surface of the bio-composites was so significantly degraded that it was impossible to determine the contact angle. During the test, a drop of measuring liquid immediately spread on the surface or penetrated the holes and cracks created during the process.

## Figures and Tables

**Table 1:** Epoxy-polyurethane compositions subjected to biodegradation process.

Epoxy-Polyurethane Composition	Polyaddition Product	Filler [wt. %]	Curing Agent
ESBO_BPA	ESBO_BPA polyaddition product of epoxidized soybean oil and bisphenol A EV = 0.118 mol/100 g HV = 144 mg KOH/g $\bar{M_{n}}$ = 1668 g/mol $\bar{M_{w}}$ = 3981 g/mol	-	Desmodur VL (based on 4,4′-diphenylmethane diisocyanate, MDI)
ESBO_BPA2%WF		2 wt.% unmodified wood waste	
ESBO_BPA5%WF		5 wt.% unmodified wood waste	
ESBO_BPA2%WF-5%NaOH		2 wt.% mercerized (with 5% solution of NaOH) wood waste	
ESBO_BPA5%WF-5%NaOH		5 wt.% mercerized (with 5% solution of NaOH) wood waste	
ESBO_BPA2%WF-10%NaOH		2 wt.% mercerized (with 10% solution of NaOH) wood waste	
ESBO_BPA5%WF-10%NaOH		5 wt.% mercerized (with 10% solution of NaOH) wood waste	

**Table 2:** List of studied enzymatic degradation conditions.

Parameter	Conditions		
	I	II	III
Incubation solution	PB1 –Na_2_HPO_4_/KH_2_PO_4_	PB2 –mixture buffer solution	PB3 –Na_2_HPO_4_/KH_2_PO_4_
Temperature	37 °C	30 °C	40 °C
Time of degradation	28 days	28 days	28 days
Amount of enzyme per sample	0.5 mg	0.5 mg	3.1 mg
Enzyme	*Porcine Pancreas/Rhizopus Oryzae*	*Porcine Pancreas/Rhizopus Oryzae*	*Porcine Pancreas*

## Data Availability

Data available on request.
